# The efficacy of a novel zinc-containing desensitizer CAREDYNE Shield for cervical dentin hypersensitivity: a pilot randomized controlled trial

**DOI:** 10.1186/s12903-022-02324-w

**Published:** 2022-07-17

**Authors:** Takashi Matsuura, Megumi Mae, Masayuki Ohira, Yumiko Mihara, Yasunori Yamashita, Kouji Sugimoto, Shizuka Yamada, Atsutoshi Yoshimura

**Affiliations:** 1 Department of Periodontology and Endodontology, Nagasaki University Graduate School of Biomedical Sciences, Nagasaki, Japan; 2 Oral Management Center, Nagasaki University Hospital, 1-7-1, Sakamoto, Nagasaki, Nagasaki 852-8588 Japan

**Keywords:** Cervical dentin hypersensitivity, Desensitizer, CAREDYNE Shield, Nanoseal, Randomized clinical trial

## Abstract

**Background:**

Recently, a novel zinc-containing desensitizer, CAREDYNE Shield, was developed. This new type of desensitizer induces chemical occlusion of dentinal tubules for desensitization and releases zinc ion for root caries prevention. Despite these features, its clinical effectiveness in the improvement of cervical dentine hypersensitivity remains to be elucidated. Thus, we aimed to evaluate the effectiveness of CAREDYNE Shield in patients with CDH.

**Methods:**

Forty CDH teeth which matched the eligibility criteria were randomly allocated to two groups in a 1:1 ratio: the CAREDYNE Shield group (intervention group) and the Nanoseal group (control group). The pain intensity in response to air stimuli, gingival condition, and oral hygiene status of CDH teeth were assessed before and at 4 weeks after treatment. The primary outcome was the reduction of pain intensity in response to air stimuli from baseline to 4 weeks after intervention.

**Results:**

From November 2019 to April 2021, 24 participants with 40 teeth were enrolled in this study and 33 teeth in 20 participants were assessed at 4 weeks after treatment. A significant reduction of pain in response to air stimuli was observed in both groups; however, no significant difference was observed between the groups.

**Conclusions:**

This study showed that CAREDYNE Shield is effective for CDH and its effectiveness is similar to Nanoseal.

***Trial registration*:**

UMIN Clinical Trials Registry (UMIN-CTR), UMIN000038072. Registered on 21st September 2019, https://center6.umin.ac.jp/cgi-open-bin/ctr/ctr_view.cgi?recptno=R000043331

**Supplementary Information:**

The online version contains supplementary material available at 10.1186/s12903-022-02324-w.

## Background

Cervical dentin hypersensitivity (CDH) is a condition wherein Individuals experience short, sharp pain in response to particular stimuli (i.e., thermal, tactile, chemical, evaporative or osmotic stimuli) that is not associated with any other dental defect or dental pathology [1, 2]. In a recent systematic review, the prevalence of CDH in various population was reported to range from 1.3 to 92.1%, and a random-effects meta-analysis estimated the prevalence of CDH was 33.5% (95% confidence interval 30.2–36.7%) [3]. The pain associated with CDH is a source of discomfort and decreases a patient’s quality of life [4]. The dentin is normally covered with enamel or cementum and is protected from external stimuli. However, non-carious cervical loss like erosion, abrasion, and abfraction causes loss of the protection. Upon exposure of the dentinal tubules to the oral environment, painful symptoms occur in response to external stimuli. For this reason, occlusion of exposed dentinal tubules is considered an ideal treatment for various agents, including resin, adhesive, glass ionomer cement, sodium fluoride varnish and potassium oxalate gels, have been investigated as potential in-office treatments for CDH [5–8].

Recently, CAREDYNE Shield (GC Dental Industrial Corporation, Tokyo, Japan), a novel zinc-containing desensitizer, was developed. This desensitizer releases not only calcium and fluorine ions for chemical occlusion of dentinal tubules but also zinc ions. Zinc ions reportedly reduce enamel and dentin demineralization, as well as inhibit plaque growth, biofilm formation, and dentin collagen degradation [9–11]. CAREDYNE Shield is therefore expected to function both as a desensitizer and as an inhibitor of root caries. Furthermore, CAREDYNE shield is biocompatible and only reacts with tooth substances. For these reasons, it can be casually applied to proximal surfaces and subgingival areas. Despite these features, its clinical effectiveness in the improvement of CDH remains to be elucidated. Thus, the present study aimed to evaluate the effectiveness of CAREDYNE Shield in the treatment of CDH in comparison to Nanoseal (Nippon Shika Yakuhin, Yamaguchi, Japan), which is a desensitizer that is commonly used in Japan. It also acts as a desensitizer by releasing calcium and fluorine ions for occlusion of dentinal tubules. Table 1 shows the PICO question of this study.

**Table 1:** PICO question

Criteria	Description
P (Participants)	Non-carious human permanent teeth with CDH
I (Intervention)	CDH treatment with CAREDYNE Shield
C (Control)	CDH treatment with Nanoseal
O (Outcome)	The reduction of pain level in response to air stimuli

*CDH* Cervical dentin hypersensitivity

## Methods

### Study design

The present pilot study was undertaken because no previous clinical studies have evaluated the efficacy of CAREDYNE Shield in improving CDH. The full study protocol was approved by the Clinical Research Ethics Committee prior to participant recruitment, registered with the University Hospital Medical Information Network-Clinical Trials Registry (UMIN-CTR; No. UMIN000038072) on September 21, 2019, and published in *Trials* on June 3, 2020 [12]. This study is a single-center, two-arm, parallel, pilot randomized controlled trial. This study protocol was developed following The Consolidated Standards of Reporting Trials (CONSORT) guidelines [13]. A CONSORT checklist is attached in Additional file 1.

### Sample size

There have been no clinical trials that can be used for sample size calculation. Thus, a minimum of 15 participants will be required in each group to perform a sample size calculation [14]. With a 20% dropout rate, a total of 40 participants will be recruited in this study.

### Enrolment

When a patient with a tooth with CDH presented to Nagasaki University Hospital, the dentist in charge of the patient assessed the eligibility criteria (Table 2). When the patient was eligible for inclusion in the study, a blinded examiner obtained their informed consent, enrolled the patient in the study, and performed baseline assessments. Then, using sealed opaque envelopes, the dentist in charge of the patient randomly allocated the patient, at a ratio of 1:1, to the intervention group (teeth with CDH were treated with CAREDYNE Shield) or the control group (teeth with CDH were treated with Nanoseal). Four operators performed the intervention treatments. The participants of the present study were blinded. The desensitizing agents used in the present study are listed in Table 3.

**Table 2:** Eligibility criteria

*Inclusion criteria*
Outpatients
Participants who presented with a complaint of CDH
Participants who agreed to attend this study after giving their informed consent
*Exclusion criteria*
Participants allergic to the desensitizing materials used in this study
Participants who are pregnant or lactating
Participants taken CDH treatment within the last 6 months
Participants with systemic disease that would mislead the results of this study
Participants who presented with pain complaints that would mislead the results of this study
CDH teeth with restoration that would mislead the results of this study
CDH teeth with caries or advanced periodontal disease
CDH teeth taken periodontal surgery or orthodontic treatment within the last 3 months

CDH: cervical dentin hypersensitivity

**Table 3:** Desensitizing agents used in this study

Material	Manufacturer	Composition
CAREDYNE Shield	GC Dental Industrial Corporation, Tokyo, Japan	Solution A: Fluorozinccalciumsilicate glass Solution B: 10–15% phosphoric acid
Nanoseal	Nippon Shika Yakuhin Co., Ltd., Shimonoseki, Japan	Solution A: Fluoroaluminocalciumsilicate glass Solution B: 10% phosphoric acid

### Intervention and clinical assessments

The target areas were cleaned, isolated using cotton rolls, and dried using cotton pellets. Then the allocated agents—mixed in two equal proportions of solution A and solution B—were applied for 20 s. Finally, the area was rinsed with water. At 4 weeks after treatment, clinical assessments of each patient were performed by the blinded examiner who enrolled them in the study.

The primary outcome of the present study was the reduction in pain in response to air stimuli at 4 weeks after intervention (vs. baseline). Pain intensity was measured using a verbal response scale (VRS [numerical scale of 0–4]). The VRS is summarized in Table 4. The change in the gingival condition near the target area (as evaluated by the gingival index [GI]) at 4 weeks after intervention (vs. baseline) and the change in the oral hygiene status of the treated dentin surface (as evaluated using a plaque index [PI] at 4 weeks after intervention (vs. baseline) were assessed as secondary to investigate the effect of Zinc ions released from CAREDYNE Shield [15, 16]. The clinical assessments were performed by two examiners, with the same examiner assessing the patient at baseline and at 4 weeks after intervention. Before the initiation of the trial, calibration was performed to promote data quality.

**Table 4:** Verbal rating scale

Score	Level of pain intensity
0	No pain
1	Mild pain
2	Moderate pain
3	Severe pain
4	Extremely intense pain

As the COVID-19 pandemic led to delayed participant recruitment and difficulty in completing this study, the study protocol was changed on October 2020. First, the recruitment period was extended by 12 months. Second, before the protocol was changed, one tooth per participant was included in this study; meanwhile, in the updated protocol, a maximum four teeth per participant (one tooth in each of the four quadrants) were included. A tooth adjacent to the treated tooth was excluded in this study.

### Statistical analysis

Fisher's exact test was used to analyze the primary outcome and the comparison of baseline characteristics that were expressed as categorical variables. The Mann–Whitney U test was used for the comparison of baseline characteristics that were expressed as continuous variables. The reduction of the pain level in response to air stimuli, GI and PI at baseline and at 4 weeks after treatment were analyzed using the Wilcoxon signed-rank test. The analyses of the present study were conducted in accordance with the intention-to-treat principle. Patients who discontinued treatment or whose treatment deviated from the study protocol, and patients with any missing data were excluded from the analysis.

## Results

**Fig. 1:**
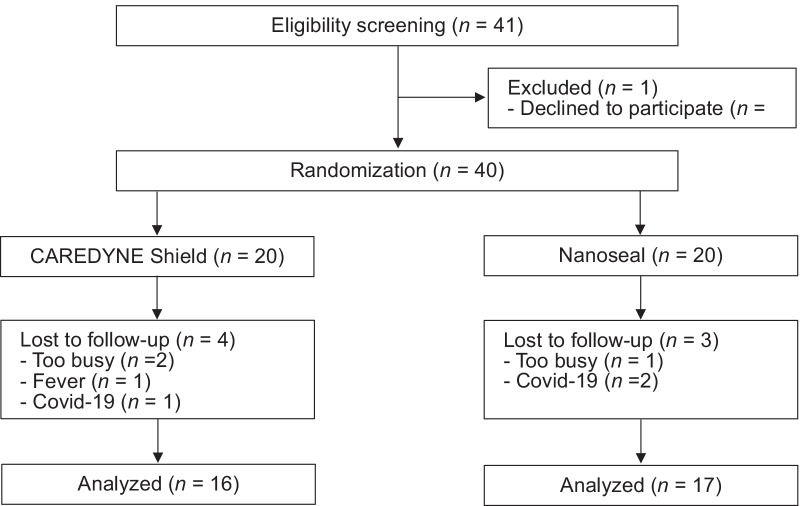
CONSORT flow diagram. From November 2019 to April 2021, 25 participants (41 teeth) matched the eligibility criteria, and 24 participants (40 teeth) who agreed to participate were enrolled in this study. At four weeks after intervention, 20 participants (33 teeth) were assessed and 4 participants (7 teeth) were lost to follow-up due to the following reasons: busy work schedule (three teeth), Covid-19 (three teeth) and fever (one tooth)

Figure 1 shows a flow diagram of this study. From November 2019 to April 2021, 25 participants (41 teeth) matched the eligibility criteria, and 24 participants (40 teeth) who agreed to participate were enrolled in this study. Every participant eligible for inclusion was included. At four weeks after intervention, 20 participants (33 teeth) were assessed and 4 participants (7 teeth) were lost to follow-up due to the following reasons: busy work schedule (three teeth), Covid-19 (three teeth) and fever (one tooth).

**Fig. 2:**
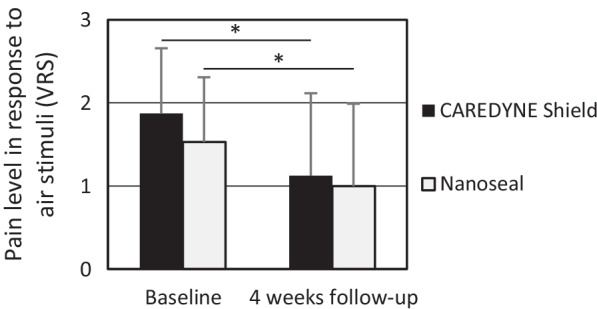
Pain level in response to air stimuli before treatment and 4 weeks after treatment. A significant reduction of the pain level in response to air stimuli was observed after treatment in both groups (*P < 0.05)

Table 5 shows the baseline characteristics of the participants who were analyzed in this study. No significant differences were observed in age, sex, or tooth type between two groups. Table 6 shows outcomes at baseline and 4 weeks after intervention. No significant differences were observed between the two groups in the pain level in response to air stimuli, GI or PI at baseline. In both groups, a significant reduction of the pain level in response to air stimuli was observed after treatment (*P* < 0.05; Fig. 2); however, no statistically significant difference was observed between two groups (*P* = 0.829). Meanwhile, no significant reduction of GI or PI was observed after treatment. And no harm was observed for any participant in this study.

**Table 5:** Baseline characteristics of the participants analyzed in this study

Variable	CAREDYNE Shield (n = 16)	Nanoseal (n = 17)	*P* value
*Age, y*			
Mean (SD)	70.6 (9.1)	63.8 (11.8)	*P* = 0.102
Range	48–80	36–80	
*Sex, n/N (%)*			
Male	2/16 (12.5%)	2/17 (11.8%)	*P* = 1.000
Female	14/16 (87.5%)	15/17 (88.2%)	
*Tooth type, n/N (%)*			
Incisor or canine	7/16 (43.7%)	8/17 (47.1%)	*P* = 1.000
Premolar	4/16 (25%)	5/17 (29.4%)	
Molar	5/16 (31.3%)	4/17 (23.5%)	
Maxillary tooth	8/16 (50%)	10/17 (58.8%)	*P* = 0.611
Mandibular tooth	8/16 (50%)	7/17 (41.2%)

**Table 6:** Outcomes at baseline and follow-up

Variable [mean (SD)]	CAREDYNE Shield (n = 16)	Nanoseal (n = 17)	*P* value
*Pain level in response to air stimuli*			
Before treatment (A)	1.88 (0.78)	1.53 (0.70)	*P* = 0.245
4 weeks after treatment (B)	1.13 (0.99)	1.00 (0.69)	
B–A	− 0.75 (1.15)	− 0.53 (0.78)	*P* = 0.829
*GI*			
Before treatment (A)	0.88 (0.86)	0.47 (0.70)	*P* = 0.217
4 weeks after treatment (B)	0.75 (0.66)	0.59 (0.60)	
B–A	− 0.13 (0.86)	0.12 (0.90)	*P* = 0.438
*PI*			
Before treatment (A)	0.50 (0.79)	0.18 (0.38)	*P* = 0.309
4 weeks after treatment (B)	0.63 (0.60)	0.24 (0.55)	
B–A	0.13 (0.86)	0.06 (0.42)	*P* = 0.335

Table 7 shows the results of subgroup analyses by tooth type or gender in the pain level in response to air stimuli. No significant differences were observed between the two groups at a reduction of the pain level in all subgroups. And no significant differences were also observed between the two groups at baseline in most subgroups. However, VRS score in CAREDYNE Shield group was significantly higher than that in Nanoseal group at baseline in mandibular tooth subgroup.

**Table 7:** Subgroup analyses of pain level in response to air stimuli by tooth type or gender

Variable [mean (SD)]	CAREDYNE Shield	Nanoseal	P value
*Incisor or canine*			
Before treatment (A)	2.14 (0.83)	1.50 (0.71)	*P* = 0.189
4 weeks after treatment (B)	1.29 (0.88)	0.63 (0.70)	
B–A	− 0.86 (1.12)	− 0.88 (0.60)	*P* = 0.608
*Premolar*			
Before treatment (A)	1.50 (0.50)	1.60 (0.80)	*P* = 1.000
4 weeks after treatment (B)	1.50 (1.12)	1.40 (0.49)	
B–A	0.00 (1.41)	− 0.20 (0.75)	*P* = 1.000
*Molar*			
Before treatment (A)	1.80 (0.69)	1.50 (0.50)	*P* = 0.730
4 weeks after treatment (B)	0.60 (0.80)	1.25 (0.43)	
B–A	− 1.20 (0.40)	− 0.25 (0.83)	*P* = 0.167
*Maxillary tooth*			
Before treatment (A)	1.50 (0.50)	1.80 (0.75)	*P* = 0.515
4 weeks after treatment (B)	1.13 (0.93)	1.10 (0.70)	
B–A	− 0.38 (0.86)	− 0.70 (0.78)	*P* = 0.342
*Mandibular tooth*			
Before treatment (A)	2.25 (0.83)	1.14 (0.35)	*P* = 0.029
4 weeks after treatment (B)	1.13 (1.05)	0.86 (0.64)	
B—A	− 1.13 (1.27)	− 0.29 (0.70)	*P* = 0.119
*Male*			
Before treatment (A)	2.00 (0.00)	1.00 (0.00)	*P* = 0.333
4 weeks after treatment (B)	0.00 (0.00)	1.00 (0.00)	
B–A	− 2.00 (0.00)	− 0.00 (1.00)	*P* = 1.000
*Female*			
Before treatment (A)	1,86 (0.83)	1.60 (0.71)	*P* = 0.621
4 weeks after treatment (B)	1.29 (0.96)	1.00 (0.63)	
B–A	− 0.57 (1.12)	− 0.60 (0.71)	*P* = 0.876

## Discussion

The present study was associated with some limitations. First, the operators were not blinded to the allocation, which possibly introduced a performance bias. This bias is unavoidable due to the nature of this study. Second, the outcome assessments were performed by two evaluators. A calibration meeting was held before starting the trial in order to improve the quality of the data. Third, intervention treatments were performed by four operators. An education meeting was held before starting the trial in order to reduce variation among operators. Fourth, in both groups, more than 80% of the study participants were female. Some studies have reported sex differences in the response to pain treatment [17]. However, this may not be unreasonable, considering that a large random sampling survey by Ye et al. reported that the male: female ratio of patients with CDH was 1:1.5, which was lower than the ratio of our study participants [18]. This may be because our study was performed from 09:00 to 17:00 on weekdays, when it was difficult for daytime workers to participate. Therefore, the external validity of this study is limited.

This study showed that CAREDYNE Shield was effective for CDH, and its effectiveness was similar to Nanoseal. However, the amount of reduction of pain intensity in response to air stimuli was lower than that in a previous study [19]. This may be because participants with more than one VRS score (0–4) were recruited in this study; meanwhile, participants with a Visual Analog Scale (VAS 0–10 cm) of > 5 cm were recruited in the previous study. And subgroup analyses indicated that VRS score in CAREDYNE Shield group was significantly higher than that in Nanoseal group at baseline in mandibular tooth subgroup (*P* = 0.029). Small sample size may cause the error; therefore, large-scale RCTs are required.

CAREDYNE Shield releases Zinc ions and is expected to reduce GI and PI [20–22]; however, no significant reduction of GI or PI was observed after treatment. This may be because the mean baseline GI and PI scores were quite low. To evaluate the effectiveness of CAREDYNE Shield for root caries inhibition, patients with low PI scores should be excluded. Therefore, we are planning to conduct another clinical trial with the recruitment of patients with high PI scores in order to evaluate the effectiveness of CAREDYNE Shield for the inhibition of root caries.

## Conclusion

In conclusion, this study showed that CAREDYNE Shield was effective for CDH and that its effectiveness was similar to Nanoseal.

## Supplementary Information

**Additional file1**. CONSORT Check List. This study protocol was developed following CONSORT guidelines

**Additional file2**. The data in this study is attached in Additional file 2

## Data Availability

The data in this study is attached in Additional file 2.

## Abbreviations

CONSORT: Consolidated Standards of Reporting Trials
CDH: Cervical dentin hypersensitivity
UMIN-CTR: The University Hospital Medical Information Network-Clinical Trials Registry
